# Aflatoxin exposure in pregnant women of mixed status of human immunodeficiency virus infection and rate of gestational weight gain: a Ugandan cohort study

**DOI:** 10.1111/tmi.13457

**Published:** 2020-07-26

**Authors:** Jacqueline M. Lauer, Barnabas K. Natamba, Shibani Ghosh, Patrick Webb, Jia‐Sheng Wang, Jeffrey K. Griffiths

**Affiliations:** ^1^ Division of Gastroenterology, Hepatology and Nutrition Boston Children’s Hospital Boston MA USA; ^2^ Feed the Future Innovation Lab for Nutrition Tufts University Boston MA USA; ^3^ MRC/UVRI and LSHTM Uganda Research Unit Noncommunicable Diseases Theme Entebbe Uganda; ^4^ Gerald J. and Dorothy R. Friedman School of Nutrition Science and Policy Tufts University Boston MA USA; ^5^ Department of Environmental Health Science University of Georgia Athens GA USA; ^6^ Department of Public Health and Community Medicine Tufts University School of Medicine Boston MA USA

**Keywords:** aflatoxin, HIV, pregnancy outcomes, Uganda, aflatoxine, VIH, résultats de la grossesse, Ouganda

## Abstract

**Objectives:**

To examine the association between aflatoxin (AF) exposure during pregnancy and rate of gestational weight gain (GWG) in a sample of pregnant women of mixed HIV status in Gulu, northern Uganda.

**Methods:**

403 pregnant women were included (133 HIV‐infected on antiretroviral therapy (ART), 270 HIV‐uninfected). Women’s weight, height and socio‐demographic characteristics were collected at baseline (~19 weeks’ gestation); weight was assessed at each follow‐up visit. Serum was collected at baseline and tested for aflatoxin B_1_‐lysine adduct (AFB‐lys) levels using high‐performance liquid chromatography (HPLC). Linear mixed‐effects models were used to examine the association between AFB‐lys levels and rate of GWG.

**Results:**

AFB‐lys levels (detected in 98.3% of samples) were higher among HIV‐infected pregnant women than HIV‐uninfected pregnant women [median (interquartile range): 4.8 (2.0, 15.0) *vs*. 3.5 (1.6, 6.1) pg/mg of albumin, *P* < 0.0001]. Adjusting for HIV status, a one‐log increase in aflatoxin levels was associated with a 16.2 g per week lower rate of GWG (*P* = 0.028). The association between AFB‐lys and the rate of GWG was stronger and significant only among HIV‐infected women on ART [−25.7 g per week per log (AFB‐lys), *P* = 0.009 for HIV‐infected women *vs*. −7.5 g per week per log (AFB‐lys), *P* = 0.422 for HIV‐uninfected women].

**Conclusions:**

Pregnant women with higher levels of AF exposure had lower rates of GWG. The association was stronger for HIV‐infected women on ART, suggesting increased risk.

## Abstract (fr)

**Objectifs:**

Examiner l'association entre l'exposition à l'aflatoxine (AF) pendant la grossesse et le taux de gain de poids pendant la grossesse (GWG) dans un échantillon de femmes enceintes de statut mixte VIH à Gulu, dans le nord de l' Ouganda.

**Méthodes:**

403 femmes enceintes ont été incluses (133 infectées par le VIH sous traitement antirétroviral (ART), 270 non infectées par le VIH). Le poids, la taille et les caractéristiques sociodémographiques des femmes ont été collectés au départ (~19 semaines de gestation); le poids a été évalué à chaque visite de suivi. Le sérum a été recueilli au départ et testé pour les niveaux d'adduit d'aflatoxine B_1_‐lysine (AFB‐lys) en utilisant la chromatographie liquide à haute performance (HPLC). Des modèles linéaires d'effets mixtes ont été utilisés pour examiner l'association entre les niveaux de AFB‐lys et le taux de GWG.

**Résultats:**

Les niveaux de AFB‐lys (détectés dans 98,3% des échantillons) étaient plus élevés chez les femmes enceintes infectées par le VIH que chez celles enceintes non infectées par le VIH [médiane (intervalle interquartile): 4,8 (2,0, 15,0) vs 3,5 (1,6, 6,1) pg/mg d'albumine, *P* <0,0001]. En ajustant pour le statut VIH, une augmentation d'un log des niveaux d'aflatoxine était associée à un taux de GWG inférieur de 16,2 g par semaine (*P* = 0,028). L'association entre AFB‐lys et le taux de GWG était plus forte et significative seulement chez les femmes infectées par le VIH sous ART [‐25,7 g par semaine et par log (AFB‐lys), *P* = 0,009 pour les femmes infectées par le VIH contre ‐7,5 g par semaine et par log (AFB‐lys), *P* = 0,422 pour les femmes non infectées par le VIH].

**Conclusions:**

Les femmes enceintes présentant des niveaux plus élevés d'exposition à l’AF avaient des taux de GWG plus faibles. L'association était plus forte pour les femmes infectées par le VIH sous ART, ce qui suggère un risque accru.

## Introduction

Gestational weight gain (GWG), the change in a pregnant woman’s weight between the beginning and the end of pregnancy, is a basic indicator of maternal and foetal health during the prenatal period [1]. Inadequate GWG is a recognised risk factor for infants being born preterm (<37 weeks gestation) and/or low birthweight (LBW) (<2500 g) particularly in low‐ and middle‐income countries (LMICs) [2, 3]. While suboptimal GWG is thought to reflect poor nutritional status, both before conception and during pregnancy, other mechanisms underlying inadequate GWG remain poorly understood [4, 5, 6].

Aflatoxins (AF) are naturally occurring, toxic metabolites of *Aspergillus* moulds, widespread in the global staple food supply (e.g. rice, corn (maize), cassava, nuts, peanuts (groundnuts), chilies and spices among many others), notably in LMICs where it is estimated that 4.5 billion people are chronically exposed [7]. Across animal and human models, AF exposure has been shown to inhibit protein synthesis [8]; promote an inflammatory response [9, 10]; and underlie a number of carcinogenic, teratogenic and immunotoxic health effects [11]. Aflatoxin B_1_ (AFB_1_), the most prevalent and toxic variety of AFs [12, 13], is a known potent class 1 human liver carcinogen and is synergistic with hepatitis B and C infections in causing liver cancer [14, 15]. Furthermore, in several studies, AFB_1_ has been linked to poor linear growth among children in LMICs [16, 17, 18], though the quality of evidence for this association remains low [19].

It is established that AFs are capable of crossing the placental barrier during pregnancy [20, 21] and are toxic to rapidly growing normal cells, as are found *in utero* and in infancy [22]. Animal studies consistently demonstrate significant adverse effects (i.e. decreased foetal weight, crown–rump length and organ weight) of AF exposure on foetal growth [23, 24, 25, 26]. A recent study found the mutagenic effect of *in utero* AF exposure in 14‐day‐old C57BL/6 mouse embryos was 20‐fold greater than in parallel‐dosed adult mice [27]. While there are relatively few human studies examining the relationship between AF exposure and foetal outcomes, a limited number of studies have demonstrated an association between maternal exposure and lower infant birthweight, small for gestational age [28, 29, 30, 31, 32] and subsequent stunted growth [33].

Proposed mechanisms by which maternal AF exposure could cause adverse foetal outcomes, including decreased GWG, include (i) induction of environmental enteric dysfunction (EED) characterised by intestinal inflammation, impaired barrier function and systemic immune activation; (ii) upregulation of pro‐inflammatory cytokines and/or downregulation of anti‐inflammatory cytokines; (iii) toxic effects on maternal and foetal organs causing systemic immune activation and impaired placental and foetal development; and (iv) impairment of the insulin‐like factor growth axis [34]. There is evidence to suggest that maternal HIV infection may exacerbate this relationship. In Ghana, HIV infection was found to be associated with higher AF levels [35, 36], speculatively due to HIV’s ability to impair liver function resulting in a decreased ability to detoxify toxic metabolites, including AF [37]. Furthermore, high AF levels were shown to accentuate some HIV‐associated changes in T‐cell phenotypes and in B cells in HIV‐infected individuals [38].

To date, we are aware of no studies which have examined the effects of AF exposure on maternal GWG, both in the general population and in those with HIV. The primary objective of this study was to examine the association between aflatoxin exposure during pregnancy (assessed via serum levels of the AFB‐lys adduct at ~19 weeks of gestation) and the rate of GWG in pregnant women of mixed HIV status in Gulu, northern Uganda.

## Methods

### Study design

Data for this study were collected from 2012 to 2013 as part of the Prenatal Nutrition and Psychosocial Health Outcomes (PreNAPs) study. PreNAPs was an observational, longitudinal cohort study designed to explore relationships among food access, nutritional and psychosocial exposures, and several physical and mental health outcomes in a sample of 403 HIV‐infected (*n* = 133) and HIV‐uninfected (*n* = 270) pregnant women in Gulu, northern Uganda. The study was approved by Cornell University’s Institutional Review Board (IRB), Gulu University’s Institutional Review Committee and the Uganda National Council for Science and Technology (UNCST). Written informed consent was obtained from all participants prior to enrolment. The parent study was registered at ClinicalTrials.gov as NCT02922829.

PreNAPs methodology (i.e. recruitment process, inclusion and exclusion criteria, response rates, and reasons for refusal to participate) has been previously reported [39, 40]. Briefly, pregnant women were recruited from the antenatal clinic (ANC) of Gulu Regional Referral Hospital (GRRH; i.e. Gulu Hospital), located in Gulu, northern Uganda. HIV‐infected and HIV‐uninfected pregnant women who presented at the ANC between 10‐ and 26‐week gestation (assessed according to women’s recall of the first date of their last menstrual period), resided within 30 km of GRRH and had a known HIV status were eligible to participate in PreNAPs. Women whose HIV status was unknown were excluded from participation.

Of the 415 pregnant women asked to participate in PreNAPs, 405 accepted, while 10 refused citing lack of time. Of the 405 participating women, complete data on all the variables of interest for this analysis were available for 403 women. In PreNAPs, HIV‐infected women were oversampled in order to achieve a minimum ratio of 1 HIV‐infected: 2 HIV‐uninfected participants. The sample size for PreNAPs was designed to provide 80% power to detect a 50‐g difference in weight gain between HIV‐infected and HIV‐uninfected women at a 5% level of significance and accounting for a 10% loss to follow‐up.

### Participation

Women in PreNAPs were followed monthly throughout their pregnancy (mean ± SD prenatal visits per woman: 5.0 ± 1.1). Women were tested for HIV at the ANC of GRRH per Government of Uganda (GoU) guidelines [41]. All HIV‐infected pregnant women were participating in a GoU ART programme to prevent mother‐to‐child transmission of HIV. Antiretroviral drugs were provided to all HIV‐infected pregnant women following the GoU [41] and WHO [42] guidelines.

At enrolment, socio‐demographic data were collected for all women including age, parity (nulliparous *vs*. other), marital status (separated, divorced or widowed *vs*. other) and education level (primary level or lower *vs*. higher than primary level). Maternal height and weight were measured at enrolment (Seca 206 for height; Seca 874 for weight; Seca North America, Chino, CA, USA). Weight was re‐assessed at all follow‐up visits. Gestational age of the index pregnancy was determined using women’s recall of the first date of their last menstrual period. Rate of GWG was calculated by dividing the difference of maternal weight between the last and first monthly visit by the corresponding difference of gestational weeks. Dietary diversity was assessed using the Minimum Dietary Diversity for Women (MDD‐W) indicator [43]. Finally, women were asked about possession of 20 household assets contained in the socio‐economic module of the 2009–2010 Uganda National Panel Survey Questionnaire [44]. An asset index was generated using principal components analysis methodology [45].

### Analysis of aflatoxin exposure levels

Serum samples were temporarily stored at −20 °C in Gulu, northern Uganda, and then transferred to Kampala, Uganda, where they were stored at −80 °C. Samples were then shipped to the laboratory at the University of Georgia, Athens, GA, USA, for analysis. AF exposure was assessed via serum levels of the AFB‐lys adduct using previously described high‐performance liquid chromatography (HPLC)‐fluorescence methods [46, 47]. This included measurement of albumin and total protein concentrations for each sample, digestion with protease to release amino acids, concentration and purification of the AFB‐lysine adduct, and finally separation and quantification by HPLC.

### GIS cluster and outlier analysis

Household geographic coordinates were obtained for 150 women in the sample. Women’s households were then mapped using geographic information system (GIS) software (ArcGIS, Esri, Redlands, CA, USA) according to HIV status (infected *vs*. uninfected) as well as serum AFB‐lys level. AF exposure data were grouped into five ranges of values determined by the natural breaks (jenks) classification method [48]. The Anselin Local Moran's *I* statistic tool [49] was used to determine whether there were statistically significant hot spots, cold spots and/or cluster outliers of HIV infection or AF levels using an inverse distance relationship.

### Statistical analysis

The main objective of this study was to determine the effect of AF exposure on the rate of GWG in a sample of pregnant women of mixed HIV status. Due to their skewed distribution, AFB‐lys levels were log‐transformed prior to analyses. Baseline characteristics for mothers were calculated and are presented as mean ± SD or *n* (%). Student’s *t*‐tests were used to compare baseline characters between HIV‐infected and HIV‐uninfected women. Associations between AFB‐lys levels and baseline characteristics were assessed using either Spearman’s correlation coefficients in the case of continuous variables or Mann–Whitney tests in the case of categorical variables.

Linear mixed‐effects models were used to determine the unadjusted and HIV status‐adjusted differences in the rate of GWG per unit increase in baseline log AFB‐lys levels. Thereafter, separate models were fit for HIV‐infected women and HIV‐uninfected women to examine whether the effect of AFs on the rate of GWG differed in the two study groups. Data were analysed using Stata 15 software (StataCorp, College Station, TX, USA). For all analyses, *P* < 0.05 was considered statistically significant.

## Results

### Baseline characteristics

Baseline characteristics for women enrolled in the study and their non‐parametric associations with AFB‐lys levels are presented in Table 1. At enrolment, women were ~25 years old, ~19 weeks’ pregnant and weighed ~61 kg. The majority were previously pregnant (~76%), married or cohabitating (~86%), and residing in a peri‐urban residence (~80%).

**Table 1:** Baseline characteristics and their non‐parametric associations with AFB‐lysine levels for 403 pregnant women in Gulu, northern Uganda

Variable	Overall (*n* = 403)†	Mann–Whitney *U* or Spearman’s *ρ*, *P*‐value‡
HIV status at recruitment		
HIV‐infected	133 (33%)	*U* = −3.2, 0.001*
Gestational age and anthropometric factors		
Gestational age (weeks)	19.4 ± 3.9	*ρ* = 0.03, 0.559
Weight (kg)	60.9 ± 8.5	*ρ* = −0.06, 0.257
Height (cm)	163.2 ± 6.0	*ρ* = 0.00, 0.949
Body mass index (BMI, kg/m^2^)	18.6 ± 2.4	*ρ* = −0.06, 0.249
Socio‐demographics		
Age (years)	24.7 ± 5.1	*ρ* = 0.01, 0.883
Asset score	4.2 ± 2.0	*ρ* = −0.15, 0.003*
Diet diversity score (MDD‐W)	4.1 ± 1.2	*ρ* = −0.03, 0.510
Nulliparous	124 (30.8%)	*U* = 0.5, 0.650
Multigravida	305 (75.7%)	*U* = −0.4, 0.678
Secondary education or higher	182 (45.2%)	*U* = 1.9, 0.061
Married or cohabiting	347 (86.1%)	*U* = 1.8, 0.074
Contextual factors		
Peri‐urban residence	323 (80.2%)	*U* = −0.4, 0.652
Ever lived in an IDP camp	208 (51.6%)	*U* = −1.1, 0.285
Ever been abducted	69 (17.1%)	*U* = −2.5, 0.011*
STI history		
History of any STI	48 (11.9%)	*U* = −2.4, 0.020*

HIV, human immunodeficiency virus; IDP, internally displaced person; MDD‐W, minimum dietary diversity for women; STI, sexually transmitted infection.

† Values are mean ± SD or *n* (%).

‡ Testing the unadjusted non‐parametric association between serum AFB‐lysine levels and each of the different continuous (Spearman’s correlation) and categorical (Mann–Whitney test) variables.

* *P* < 0.05.

At baseline, HIV‐infected women in the sample were significantly different from HIV‐uninfected women with respect to mean age (25.8 *vs*. 24.1 years, *P* = 0.001), asset score (3.6 *vs*. 4.5, *P* < 0.001), diet diversity score (MDD‐W) (3.8 *vs*. 4.2, *P* = 0.003) and gestational age (18.6 *vs*. 19.8 weeks, *P* = 0.007). Additionally, HIV‐infected women in the sample were less likely to be married or cohabitating (76.7% *vs*. 90.7%, *P* < 0.001) and less educated (secondary education or higher: 35.3% *vs*. 50.0%, *P* = 0.005); they were more likely to have previously been pregnant (multigravida: 85.0% *vs*. 71.1%, *P* = 0.002) and to have been abducted in the past (24.1% *vs*. 13.7%, *P* = 0.009).

In unadjusted non‐parametric analyses, AFB‐lys levels were significantly associated with several baseline characteristics including HIV infection (*U* = −3.2, *P* = 0.001), asset score (*ρ* = −0.15, *P* = 0.003) and STI history (*U* = −2.4, *P* = 0.02).

### AFB‐lys levels

**Figure 1:**
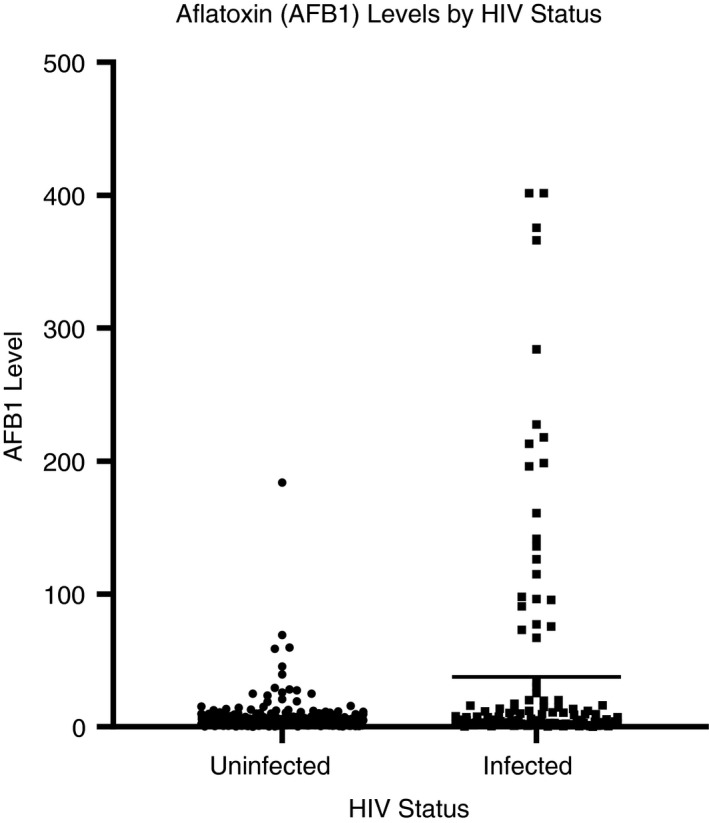
AFB‐lysine levels by HIV status for 403 women in Gulu, northern Uganda.

AFB‐lys levels were detected in 98.5% of samples [median: 3.7 pg/mg albumin (range: 0.1–401.5 pg/mg albumin, interquartile range (IQR): 1.7–7.9 pg/mg albumin)]. The arithmetic mean AFB‐lys level was 16.6 pg/mg albumin (95% confidence interval (CI): 11.6–21.6 pg/mg albumin), and the geometric mean AFB‐lys level was 4.2 pg/mg albumin (95% CI: 3.6‐4.8 pg/mg albumin). Figure 1 shows the distribution of AFB‐lys levels for HIV‐uninfected women compared with HIV‐infected women. AFB‐lys levels were significantly higher among HIV‐infected women compared with HIV‐uninfected women [median (IQR): 4.8 (2.0–15.0) *vs*. 3.5 (1.6, 6.1) pg/mg albumin, *P* = 0.001].

### Association between AF Exposure and Rate of GWG

**Figure 2:**
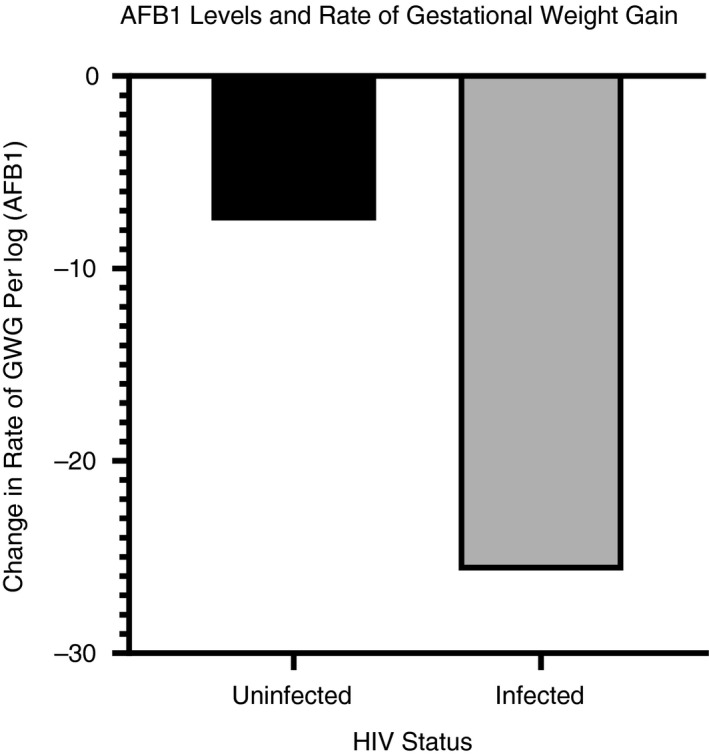
Association between a one‐log increase in AFB‐lysine levels and rate of gestational weight gain (GWG) during the second and third trimesters for 403 pregnant women in Gulu, northern Uganda.

Table 2 shows the differences in the rate of GWG per one‐log increase in AFB‐lys levels using unadjusted and adjusted linear mixed‐effects models. Adjusting for HIV status, a one‐log increase in AFB‐lys was associated with a 16.2 g per week lower rate of GWG (*P* = 0.028). Furthermore, as shown in Figure 2, a one‐log increase in AFB‐lys was associated with a larger rate of GWG in HIV‐infected women compared with HIV‐uninfected women. For every one‐log increase in AFB‐lys level, the rate of GWG in HIV‐infected women decreased by 25.7 g per week (*P* = 0.009) compared with 7.5 g per week for HIV‐uninfected women (*P* = 0.422).

**Table 2:** Linear mixed‐effects models to determine the unadjusted and covariate‐adjusted differences in the rate of gestational weight gain (GWG) per one‐log increase in AFB‐lysine levels for 403 women in Gulu, northern Uganda

Model parameter	Unadjusted model	Adjusted model†, ‡
Constant (*β*_0_), starting weight	59.0 ± 0.6 kg	58.5 ± 0.7 kg
Effect of the time variable (gestational age; *β*_1_), the rate of GWG	428.5 ± 24.9 g per week (<0.001)*	442.4 ± 24.6 g per week (<0.001)*
Effect of the quadratic term of the time variable (gestational age squared; *β*_2_)	4.1 ± 0.7 g per week^2^ (<0.001)*	4.1 ± 0.7 g per week^2^ (<0.001)*
Effect of the AFB1 exposure (*β*_3_) on starting weight	0.2 ± 0.3 kg (NS)	0.4 ± 0.3 kg (NS)
Effect of exposure on the effect of the time variable (*β*_4_), differences in the rate of GWG	(−)20.4 ± 7.1 g per week (0.004)*	(−)16.2 ± 7.4 g per week (0.028)*
Effect of exposure on the quadratic term of time variable (*β*_5_)	Not modelled	Not modelled

† Adjusted for HIV status (infected *vs*. uninfected); that is, the only variable associated with baseline AFB‐lysine levels as well as GWG.

‡ Gestational age is centred at 13 weeks; HIV‐uninfected status is the reference category.

* *P* < 0.05.

### GIS results

**Figure 3:**
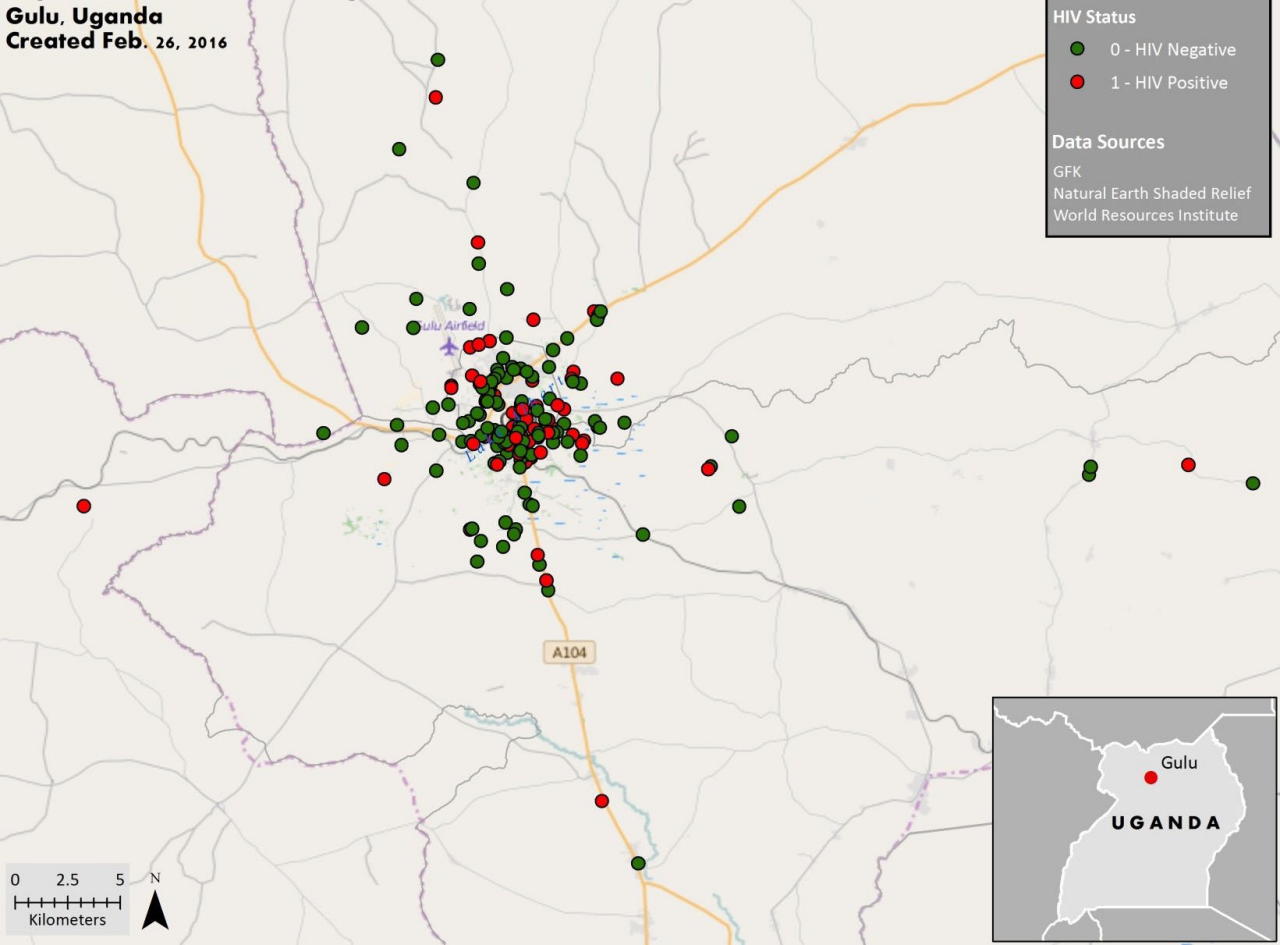
Household locations for HIV‐infected and HIV‐uninfected pregnant women (*n* = 150) in Gulu, northern Uganda. The Anselin Local Moran's I statistic tool was used to determine whether there were statistically significantly hot spots, cold spots and/or cluster outliers using an inverse distance relationship. No statistically significant findings were observed for either HIV‐infected or HIV‐uninfected women.

**Figure 4:**
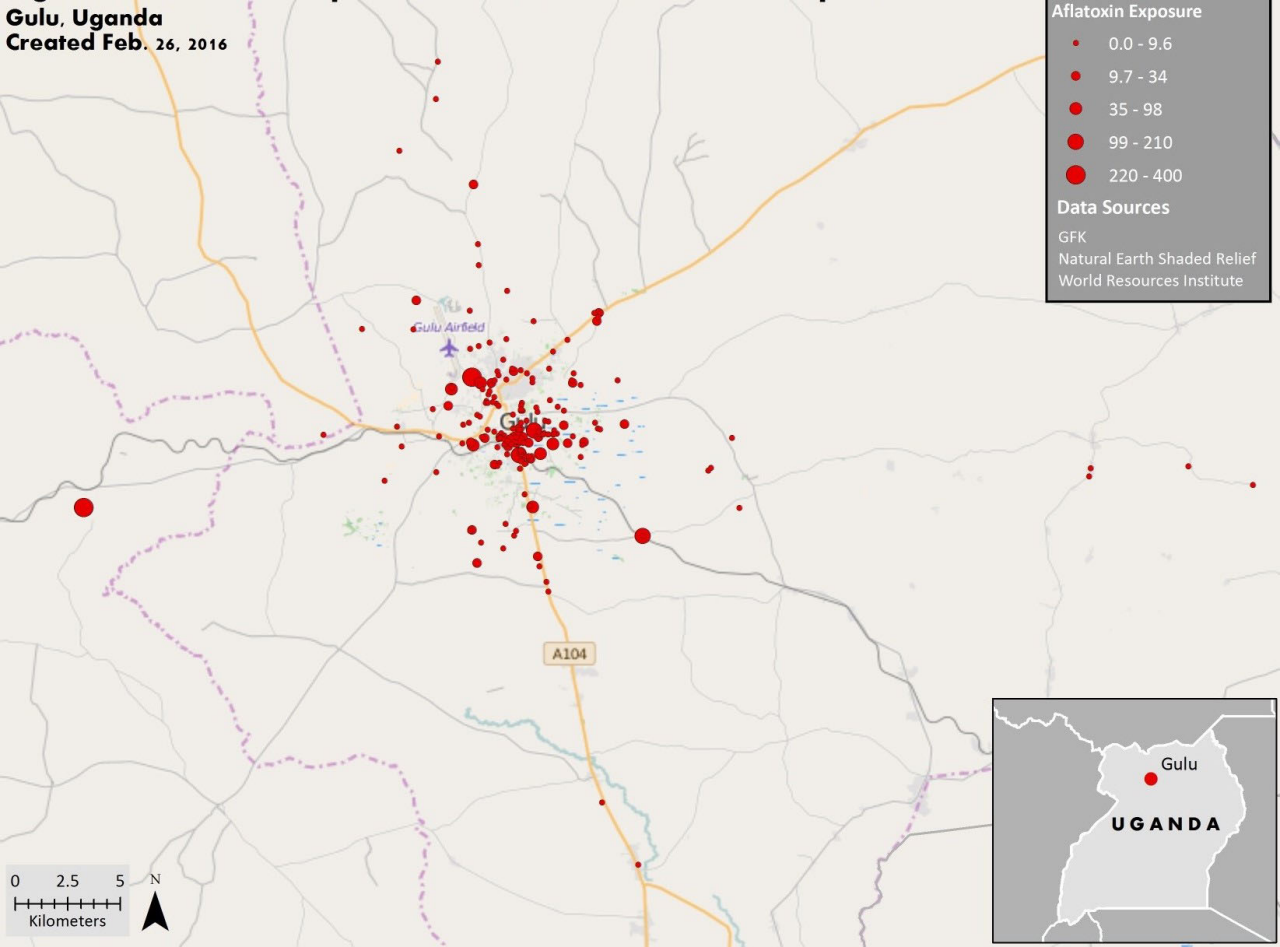
AFB‐lysine levels for HIV‐infected and HIV‐uninfected pregnant women (*n* = 150) in Gulu, northern Uganda. Exposure data were grouped into 5 ranges of values determined by the natural breaks (jenks) classification method. The Anselin Local Moran's I statistic tool was used to determine whether there were statistically significantly hot spots, cold spots and/or cluster outliers using an inverse distance relationship. Two statistically significant high outliers were observed.

No statistically significant hot spots, cold spots and/or cluster outliers were observed for either HIV‐infected or HIV‐uninfected women (Figure 3). Two statistically significant individual high outliers were observed for level of AF exposure, both of whom were HIV‐infected women (Figure 4).

## Discussion

In this study of 403 pregnant women of mixed HIV status in northern Uganda, we examined the association between AF exposure during pregnancy and rate of GWG. AF exposure (as determined by a validated and reliable exposure biomarker, AFB‐lys), in this population, was nearly universal (detected in ~98% of samples) and ranged widely from 0.1 to 401.5 pg/mg albumin. This highly skewed distribution is consistent with those from another recent study, which found that 100% of samples from 220 HIV‐negative pregnant Ugandan women had detectable AFB_1_ levels ranging from 0.71 to 95.60 pg/mg albumin [31]. Notably, however, a number of women in this study had alarmingly high levels of AF exposure with 16 women having AFB‐lys levels over 100 pg/mg albumin (15 of 16 were in the HIV‐infected group). For comparison purposes, Table S1 shows the reported levels of AF exposure from previous studies of pregnant women and infants. Standardised levels of exposure were calculated based on previously published conversion factors [50].

Furthermore, in this study we observed significantly higher AF exposure levels among HIV‐infected women compared with HIV‐uninfected women. Using GIS, we tested the hypothesis that HIV‐infected women, perhaps because of stigma or poverty, live in areas of Gulu where the markets sell food that is significantly more contaminated with AFs. We found almost no indication of spatial clustering in these analyses, suggesting this was not the case. Therefore, given our findings, we surmise that HIV infection in and of itself, or being treated for HIV with ARTs, may put pregnant women at risk of having higher AFB‐lys levels.

Because both AF exposure and HIV are known to cause immune suppression [51, 52], a synergistic relationship between the two may exist [53]. In a cross‐sectional study of 314 (155 HIV‐infected, 159 HIV‐uninfected) Ghanaian participants, Jolly *et al*. [35] observed significantly higher AFB‐albumin levels among HIV‐infected participants (mean ± SD: 1.06 ± 0.60 pmol/mg albumin) compared with HIV‐uninfected participants (mean ± SD: 0.91 ± 0.46 pmol/mg albumin). In a subsequent cross‐sectional study of 314 antiretroviral‐naïve, HIV‐positive Ghanaian participants with median CD4 count of 574 cells/μl blood (mean ± SD = 630 ± 277 cells/μl blood), it was observed that HIV viral load increased as AFB‐albumin levels increased. In the study, ‘high’ viral load was 2.3 times more likely among participants in the third AFB‐albumin quartile (95% CI: 1.13, 4.51), and 2.9 times more likely among participants in the fourth AFB‐albumin quartile (95% CI: 1.41, 5.88), compared to participants in the first quartile [36].

Results from these studies suggest that HIV‐infected, untreated individuals are more likely to have higher AF exposure levels than HIV‐uninfected individuals. Our study was conducted in a group of HIV‐infected women taking a first‐line ART regimen. Examining viral load suppression data from all samples from the Gulu hub (August 2014–June 2019) from women receiving first‐line therapy in pregnancy, 92.3% of 1385 tested samples showed viral suppression [54]. While we do not have individual viral suppression data from the women enrolled in this study, we may reasonably infer (since first‐line therapy has not changed) that the women with HIV in this study would have received therapy that led to viral suppression in approximately 92.3% of pregnant recipients seen at the Gulu regional HIV facilities.

We note that the connection between HIV infection and higher AFB‐lys levels is not well understood; however, it may speculatively be due to HIV’s ability to impair liver function resulting in a decreased ability to detoxify toxic metabolites, including AF [37]. Furthermore, in addition to the virus itself, ARVs may also play a role. To date, ARVs have been linked to various liver diseases, including hepatotoxicity and idiosyncratic liver injury, which may also contribute to the accumulation of AFB‐lys in the bloodstream [55]. Overall, AFs may act in synergy with both HIV and ARTs to damage liver function and ultimately increase exposure levels.

Finally, in this study we observed that the rate of GWG was lower among pregnant women with higher AFB‐lys levels. This association was stronger and significant only among HIV‐infected women. The results from our study are consistent with several previous studies, which have demonstrated a link between AF exposure during pregnancy and adverse foetal outcomes, particularly LBW, for which inadequate GWG is a risk factor. In a cross‐sectional study of 785 pregnant Ghanaian women with unreported HIV status, Shuaib *et al*. [32] observed that participants in the highest AFB‐albumin quartile were more likely to have infants born LBW (OR: 2.09, 95% CI: 1.19–3.68). Likewise, in a prospective cohort study of 220 HIV‐uninfected pregnant Ugandan women, Lauer *et al*. [31] observed that maternal AF exposure assessed at ~18 weeks’ gestation was associated with infants born with lower birthweight (*β*: −0.07, 95% CI: −0.13, −0.003) and smaller head circumference (*β*: −0.26, 95% CI: −0.49, −0.02).

While these studies have examined the relationship between AF exposure and infant birth outcomes, as far as we are aware, our study is the first to look at the impacts of AF exposure on maternal pregnancy outcomes, that is GWG, and to demonstrate that the association between AF exposure and adverse foetal outcomes may, in fact, be stronger for HIV‐infected women on ARTs compared with HIV‐uninfected women. There are, however, several limitations worthy of mention. Notably, gestation was assessed according to women’s recall of the first date of their last menstrual period, which is subject to error due to factors such as recall bias, irregular menses and individual variation in menstrual cycle length [56]. Furthermore, as this was an observational cohort study which prospectively assessed observed outcomes with a relatively small samples size, we have limited ability to draw conclusions regarding causality or the directionality of the relationships among AF exposure, HIV and ARTs. However, given the widespread AF exposure, large numbers of HIV‐infected individuals on ARTs and high rates of infants born LBW, further studies examining these relationships are warranted.

## Declarations

The authors declare they have no actual or potential competing financial interests. Support for this effort was provided by (i) the Feed the Future Innovation Lab for Nutrition, which is funded by the United States Agency for International Development (USAID), under the term of Contract No. AID OAA‐L‐10‐0006 via Mission Support Funding USAID/East Africa; and (ii) faculty funds to JKG from Tufts University. The opinions expressed herein are those of the authors(s) and do not necessarily reflect the views of the U.S. Agency for International Development. The authors further wish to acknowledge Dr. Sera L. Young for her contributions to the design and implementation of the PreNAPs study.

## Supporting information


**Table S1.** Aflatoxin exposure levels reported in previous studies.
