# Supplementary material for: Aflatoxin exposure in pregnant women of mixed status of human immunodeficiency virus infection and rate of gestational weight gain: a Ugandan cohort study
Source: Trop Med Int Health. 2020 Jul 26;25(9):1145–54. doi: 10.1111/tmi.13457 (PMC7539974; doi:10.1111/tmi.13457)
Supplement: Supplementary file 1 — Table S1. Aflatoxin exposure levels reported in previous studies. [file TMI-25-1145-s001.docx]

Supplemental Table 1: Aflatoxin exposure levels reported in previous studies

| **Publication Year** | **Study Site** | **Studied Groups** | **Mean, median, or geometric mean of AF exposure reported** | **Method** | **Normalized Exposure^1^** | **Comments** |
| --- | --- | --- | --- | --- | --- | --- |
| 2019 | Gulu, Uganda (This study) | 403 pregnant women (133 HIV infected on therapy, 270 HIV uninfected) ~ 19 weeks of gestation | Ranges:  All: 0.1-401.5  HIV(+):0.1-401.5  HIV(-): 0.1-183.9  Arithmetic Means (95% CI):  All: 16.6 (11.6, 21.6)  HIV (+): 37.6 (23.2, 52.0)  HIV (-): 6.5 (4.8, 8.1)  Geometric Means (95% CI):  All: 4.2 (3.6, 4.8)  HIV(+): 6.7 (4.9, 9.2)  HIV(-): 3.3 (2.9, 3.8)  Medians (IQR):  All: 3.7 (1.7, 7.9)  HIV (+): 4.8 (2.0, 15.0)  HIV (-): 3.5 (1.6, 6.1) | HPLC-f | Median (IQR):  All: 3.7 (1.7, 7.9)  HIV (+): 4.8 (2.0, 15.0)  HIV (-): 3.5 (1.6, 6.1)  pg/mg albumin | All HIV (+) women were on anti-retroviral therapy |
| 2019 | Banke, Nepal^1^ | 1621 pregnant women at 136 ± 43 (mean ± SD) days gestation | Geometric Mean (95% CI): 1.37 pg/mg albumin (1.30, 1.44 pg/mg albumin) | HPLC-f |  | Maternal AFB_1_-lysine adduct levels significantly associated with SGA (OR: 1.13; 95% CI: 1.00, 1.27) |
| 2018 | Mukono, Uganda^2^ | 258 pregnant women at 17.8 + 3.5 (mean ± SD) weeks gestation | Median: 5.83 pg/mg albumin (range: 0.71-95.60 pg/mg albumin, IQR: 3.53-9.62 pg/mg albumin). | HPLC-f | 5.71 pg/mg albumin | Maternal AFB_1_-lysine adduct levels significantly associated with lower weight/WAZ and smaller head circumference/HCZ. |
| 2017 | Nepal^3^ | 85 children aged 15, 24, 36 months. | Geometric Mean: 3.62 pg /mg albumin | IDMS | 2.43 pg/mg albumin | No association with child growth. |
| 2015 | Southwest Uganda^4^ | Archived serum from 2 cohorts  713 samples 1989 – 2010 GPC cohort  374 samples 2000-2003 RCCS cohort | GPC:  Mean 2.85 + 8.37; median 1.58  Range 0.4-168.0  HIV (+) median 3.57  HIV (-) median 1.58  RCCS:  Mean 3.46 + 9.25; median 1.18  Range: 0.4-122.5  HIV (+:) 3.94  HIV (-): 1.61 | HPLC-f | GPC: Geometric mean 1.62  Mean 2.85 + 8.37; median 1.58  Range 0.4-168.0  RCCS: Geometric mean 1.66  Mean 3.46 + 9.25; median 1.18  Range 0.4-122.5 |  |
| 2014 | Southwest Uganda^5^ | 100 adults, and 96 children under 3 years of age | Geometric Mean (95% CI):  Adults: 11.5 (10.2-13.0)  Children: 9.7 (8.2-11.5)  Range: 0 to 237.7 | ELISA | Geometric Mean (95% CI):  Adults: 1.5 (1.4-1.7)  Children: 1.3 (1.1-1.5)  Range: 0 to 31.6 | All adults, and all but 4 children had detectable AF exposure. No differences by HIV status. |
| 2014 | Gambia, West Africa^6^ | 134 women in early pregnancy and 99 in later pregnancy (8 + 4 weeks, and 27 + 3 weeks of gestation, respectively) | Early: 34.5 (29.3, 40.7 95% CI)  Later: 41.8 (34.7, 50.3 95% CI) | ELISA | Early: 4.58 (3.89, 5.41)  Later: 5.55 (4.61, 6.68) |  |
| 2014 | Nepal, Bangladesh^7^ | Archived Nepal serum from 1999-2001; from 2008-2012 Bangladesh |  |  |  |  |
|  |  | N=30 Nepalese women 1^st^ and 3^rd^ trimester | Median 18.08 1^st^ trimester; 25.35 3^rd^ trimester | IDMS | Median 12.11 1^st^ trimester  Median 16.98 3^rd^ trimester | No significant change in AF exposure across trimesters |
|  |  | N=141 Nepalese women early pregnancy | Median 22.45, geometric mean 25.28, 7.42-77.94 IQR | IDMS | Median 15.04 early pregnancy  GM 16.9, IQR 5.0-52.2 |  |
| 2013 | Qidong, China^8^ | 100 archived samples from adults 1989, 1999, 2009 | 1989: median 19.3  2009: undetectable <0.5 | IDMS |  |  |
| 2011 | Ghana^9^ | 314 adults, 155 HIV (+) and 159 HIV (-)  30% of participants on HIV anti-retroviral therapy. | Mean + SD  HIV (+): 1.06 + 0.60 range 0 - 3.48  HIV (-): 0.91 + 0.46 range 0.12 - 3.00  Median HIV (+) 0.86  Median HIV (-) 0.81 | HPLC-f | Mean + SD  HIV (+): 1.06 + 0.60 range 0 - 3.48  HIV (-): 0.91 + 0.46 range 0.12 - 3.00  Median HIV (+) 0.86  Median HIV (-) 0.81 |  |
| 2010 | Kumasi, Ghana, West Africa^10^ | Cross-sectional study of 755 pregnant women enrolled in 2006 | Mean 10.9 + 19 pg/mg albumin  Range 0.44-268.73 | HPLC-f | Mean 10.9 pg/mg albumin  Range 0.44-268.73 | Odds of anemia in women increased 21% (OR 1.21, 1.04-1.39) with each quartile of AF exposure |
|  | Kumasi, Ghana, West Africa | Association between AF exposure in 785 women and infant outcomes | Mean 10.9 + 19 pg/mg albumin  Range 0.44-268.73 | HPLC-f | Mean 10.9 pg/mg albumin  Range 0.44-268.73 | High maternal AF exposure predicted low infant birthweight (p=0.007 for trend) |
| 2007 | Gambia, West Africa^11^ | Studied influence of maternal AF in utero exposure on growth of 138 children. AF exposure measured 4.5, and 0.9, months before delivery. | AF detected in all tested samples  Authors averaged the two measures  Median 39.9 (IQR 23.3-64.1)  Geometric mean 40.4 (Range, 4.8 -260.8) | ELISA | Median 5.3 (IQR 3.1-8.5)  Geometric mean 5.4 (Range, 0.6 -34.6) | Higher maternal AF exposure levels -> stunting. 1 log increase in AF predicted 0.5 HAZ score decrease in infants |
| 2004 | Benin, West Africa^12^ | 200 children aged 16-37 months recruited from 4 villages, 50 per village | Mean, 95% CI  Bagbe: 11.8 (9.2-15.2)  Sedje: 31.1 (25.4-38.0)  Djidja: 45.9 (35.7-59.0)  Dovi-Dogbe: 119.3 (96.2-148.1) | ELISA | Mean, 95% CI  Bagbe: 1.6 (1.2-2.0)  Sedje: 4.1 (3.4-5.0)  Djidja: 6.1 (4.7-7.8)  Dovi-Dogbe: 15.8 (12.8-19.7) | Strong (p<0.0001) inverse relationship between AF exposure and height growth / stunting over 8 months |

Converted values obtained via conversion factors published in McCoy LF, Scholl PF, Sutclifee AE, et al. Human Aflatoxin Albumin Adducts Quantitatively Compared by ELISA, HPLC with Fluorescence Detection, and HPLC with Isotope Dilutin Mass Spectrometry. Cancer Epidemiol Biomarkers Prev 2008; 17:1653-1657

(HPLC-f/IDMS 0.67, ELISA / HPLC-f 7.53, ELISA / IDMS 4.99)

^1^Andrews-Trevino JY, Webb P, Shively G, Rogers BL, Baral K, Davis D, Paudel K, Pokharel A, Shrestha R, Wang JS, Ghosh S. Relatively low maternal aflatoxin exposure is associated with small-for-gestational-age but not with other birth outcomes in a prospective birth cohort study of Nepalese infants. The Journal of nutrition. 2019.

^2^Lauer JM, Duggan CP, Ausman LM, Griffiths JK, Webb P, Wang JS, Xue KS, Agaba E, Nshakira N, Ghosh S. Maternal aflatoxin exposure during pregnancy and adverse birth outcomes in Uganda. Maternal & child nutrition. 2019 Apr;15(2):e12701.

^3^Mitchell NJ, Hsu HH, Chandyo RK, Shrestha B, Bodhidatta L, Tu YK, Gong YY, Egner PA, Ulak M, Groopman JD, Wu F. Aflatoxin exposure during the first 36 months of life was not associated with impaired growth in Nepalese children: An extension of the MAL-ED study. PloS one. 2017 Feb 17;12(2):e0172124.

^4^Kang MS, Nkurunziza P, Muwanika R, Qian G, Tang L, Song X, Xue K, Nkwata A, Ssempebwa J, Lutalo T, Asiki G. Longitudinal evaluation of aflatoxin exposure in two cohorts in south-western Uganda. Food Additives & Contaminants: Part A. 2015 Aug 3;32(8):1322-30.

^5^Asiki G, Seeley J, Srey C, Baisley K, Lightfoot T, Archileo K, Agol D, Abaasa A, Wakeham K, Routledge MN, Wild CP. A pilot study to evaluate aflatoxin exposure in a rural Ugandan population. Tropical medicine & international health. 2014 May;19(5):592-9.

^6^Castelino JM, Dominguez‐Salas P, Routledge MN, Prentice AM, Moore SE, Hennig BJ, Wild CP, Gong YY. Seasonal and gestation stage associated differences in aflatoxin exposure in pregnant Gambian women. Tropical medicine & international health. 2014 Mar;19(3):348-54.

^7^Groopman JD, Egner PA, Schulze KJ, Wu LS, Merrill R, Mehra S, Shamim AA, Ali H, Shaikh S, Gernand A, Khatry SK. Aflatoxin exposure during the first 1000 days of life in rural South Asia assessed by aflatoxin B1-lysine albumin biomarkers. Food and chemical toxicology. 2014 Dec 1;74:184-9.

^8^Chen JG, Egner PA, Ng D, Jacobson LP, Muñoz A, Zhu YR, Qian GS, Wu F, Yuan JM, Groopman JD, Kensler TW. Reduced aflatoxin exposure presages decline in liver cancer mortality in an endemic region of China. Cancer prevention research. 2013 Oct 1;6(10):1038-45.

^9^Shuaib FM, Jolly PE, Ehiri JE, Jiang Y, Ellis WO, Stiles JK, Yatich NJ, Funkhouser E, Person SD, Wilson C, Williams JH. Association between anemia and aflatoxin B1 biomarker levels among pregnant women in Kumasi, Ghana. The American journal of tropical medicine and hygiene. 2010 Nov 5;83(5):1077-83.

^10^Shuaib FM, Jolly PE, Ehiri JE, Yatich N, Jiang Y, Funkhouser E, Person SD, Wilson C, Ellis WO, Wang JS, Williams JH. Association between birth outcomes and aflatoxin B1 biomarker blood levels in pregnant women in Kumasi, Ghana. Tropical Medicine & International Health. 2010 Feb;15(2):160-7.

^11^Turner PC, Collinson AC, Cheung YB, Gong Y, Hall AJ, Prentice AM, Wild CP. Aflatoxin exposure in utero causes growth faltering in Gambian infants. Int J Epidemiol 2007; 36(5): 1119-25.

^12^Gong Y, Hounsa A, Egal S, Turner PC, Sutcliffe AE, Hall AJ, Cardwell K, Wild CP. Postweaning exposure to aflatoxin results in impaired child growth: a longitudinal study in Benin, West Africa. Environmental health perspectives. 2004 Apr 27;112(13):1334-8.
